# Reducing salt intake for prevention of cardiovascular diseases in high-risk patients by advanced health education intervention (RESIP-CVD study), Northern Thailand: study protocol for a cluster randomized trial

**DOI:** 10.1186/1745-6215-13-158

**Published:** 2012-09-04

**Authors:** Myo Nyein Aung, Motoyuki Yuasa, Saiyud Moolphate, Supalert Nedsuwan, Hidehiro Yokokawa, Tsutomu Kitajima, Kazuo Minematsu, Susumu Tanimura, Hiroshi Fukuda, Yoshimune Hiratsuka, Koichi Ono, Sachio Kawai, Eiji Marui

**Affiliations:** 1 Department of Public Health, Juntendo University School of Medicine, Hongo 2-1-1, Bunkyo-ku, Tokyo 113-8421, Japan; 2 Boromarajonani College of Nursing Nakhon Lampang (BCNLP), Lampang, Thailand; 3 TB/HIV Research Consortium, Chiang Rai, Thailand; 4 Department of Social and Preventive Medicine, Chiang Rai Regional Hospital, Chiang Rai, Thailand; 5 Department of General Medicine, Juntendo University School of Medicine, Tokyo, Japan; 6 Faculty of Social Science, Kyorin University Graduate School of International Cooperation Studies, Hachioji, Japan; 7 Department of Public Health, Hyogo College of Medicine, Nishinomiya, Japan; 8 Department of Health and Welfare Services, National Institute of Public Health, Wako, Japan; 9 Department of Ophthalmology, Juntendo University School of Medicine, Tokyo, Japan; 10 Department of Sport Medicine, Juntendo University School of Health and Sport Science, Inba, Japan

**Keywords:** Salt, CVD, Thailand, Behavior, Intervention

## Abstract

**Background:**

Decreasing salt consumption can prevent cardiovascular diseases (CVD). Practically, it is difficult to promote people’s awareness of daily salt intake and to change their eating habits in terms of reducing salt intake for better cardiovascular health. Health education programs visualizing daily dietary salt content and intake may promote lifestyle changes in patients at high risk of cardiovascular diseases.

**Methods/Design:**

This is a cluster randomized trial. A total of 800 high-CVD-risk patients attending diabetes and hypertension clinics at health centers in Muang District, Chiang Rai province, Thailand, will be studied with informed consent. A health center recruiting 100 participants is a cluster, the unit of randomization. Eight clusters will be randomized into intervention and control arms and followed up for 1 year. Within the intervention clusters the following will be undertaken: (1) salt content in the daily diet will be measured and shown to study participants; (2) 24-hour salt intake will be estimated in overnight-collected urine and the results shown to the participants; (3) a dietician will assist small group health education classes in cooking meals with less salt. The primary outcome is blood pressure change at the 1-year follow-up. Secondary outcomes at the 1-year follow-up are estimated 24-hoursalt intake, incidence of CVD events and CVD death. The intention-to-treat analysis will be followed.

Blood pressure and estimated 24-hour salt intake will be compared between intervention and control groups at the cluster and individual level at the 1-year follow-up. Clinical CVD events and deaths will be analyzed by time-event analysis. Retinal blood vessel calibers of CVD-risk patients will be assessed cross-sectionally. Behavioral change to reduce salt intake and the influencing factors will be determined by structured equation model (SEM). Multilevel regression analyses will be applied. Finally, the cost effectiveness of the intervention will be analyzed.

**Discussion:**

This study is unique as it will recruit the individuals most vulnerable to CVD morbidity and mortality by applying the general Framingham CVD risk scoring system. Dietary salt reduction will be applied as a prioritized, community level intervention for the prevention of CVD in a developing country.

**Trial registration:**

ISRCTN39416277

## Background

Cardiovascular diseases (CVD) are the world’s largest killers, causing more than 17 million estimated deaths globally in 2008 [1]. Over 80% of these CVD deaths occurred in low- and middle-income countries [1]. There is an urgent need to prevent CVD morbidity and mortality in developing countries [2]. In Thailand, CVD events are currently in the top three leading causes of death [3] and leading causes of admissions to provincial and community-level hospitals [4]. Thus, prevention of CVD events in high-risk individuals would be the most cost-effective strategy for reducing the morbidity and mortality caused by CVD in low-middle-income countries such as Thailand [5].

Salt reduction at the community level is the WHO-suggested strategy for prevention of CVD in developing countries [2,6-8]. In recent years, it has become a proven, effective strategy in many countries such as Finland, Japan and some developed countries [6,9-11]. In Asia, strokes are common CVD events, with hypertension as the most attributable risk factor for CVD; consequently, salt intake is likely to be the most important risk behavior to be controlled for CVD prevention [12]. However, evidence for implementation of reducing salt intake in the developing country setting is still scarce and inadequate, especially in Southeast Asian countries [13]. The cost effectiveness of the salt reduction strategy for CVD prevention is also yet to be determined [14].

Human beings love the taste of salt, and the consumption of salty foods has become a well-entrenched dietary habit [15]. Many patients with CVD risks know that a high level of salt intake is a risky behavior. However, changing the behavior to a reduction in salt intake is not easily done in practice. It is necessary to strengthen the hearsay knowledge and relayed massage among the high-risk persons so that a strong perception will develop, leading to behavioral change [16]. “Seeing is believing” [17]. We hypothesized that visualizing the salt content in daily food and the rough amount of individual daily salt intake in high-risk persons could lead to behavioral change.

In this study, we will research intensive health education interventions that use visualization tools to make individuals aware of their daily salt consumption. The participants will be informed of the salt content in a variety of daily foods as well as their 24-hour estimated salt intake by urine sodium measurement. The health promotion strategy aims to reduce salt intake to prevent CVD. The implementation of the strategy will be centered at community health centers in Chiang Rai province in northern Thailand. It is expected to bring about behavioral changes leading to voluntary salt reduction among high-risk patients, thereby averting thousands of CVD events and deaths.

## Methods/Design

### Study objectives

The primary objective is to compare the effectiveness of health education via visualization of salt content in food, soup and urine, with routine health education by means of measuring blood pressure changes in high-CVD-risk patients at 1-year follow-up.

The secondary objectives are: to compare the effectiveness of specially designed health education with routine health education by means of monitoring estimated daily salt intake using urine sodium measurement, CVD incidence and retinal vessel caliber changes among diabetic and hypertension patients with high CVD risk. We will also compare the cost-effectiveness of health education via visualization of salt content in food, soup and urine with routine health education among diabetic and hypertensive patients with high CVD risk.

### Design

This is a cluster randomized controlled trial (CRT). The rationale for choosing a CRT design is to prevent contamination by sharing second-hand massage between participants of intervention and control arms. Instead of randomizing the individual patient, clusters of eligible patients will be randomly allocated into the intervention and control arms. There will be two arms of CRT: the salt reduction intervention arm and control arm. The units of randomization are health centers.

### Study site: clusters and characteristics of the clusters

**Figure 1:**
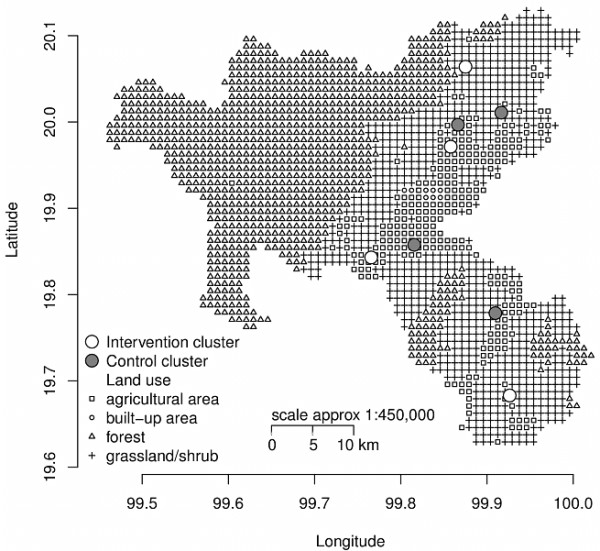
Map showing study area and clusters.

The study will be conducted in Muang district, Chiang Rai, the northernmost province of Thailand. Every health center within the study site district, which has a hypertension and diabetes clinic, will be screened for eligibility criteria. In order to prevent an empty cluster, any health center with a total of fewer than 200 patients attending the hypertension and diabetes clinic will not be included. In this study, one health center will form one cluster. Each cluster will have 100 participants, and there will be four clusters in each study arm. The locations of randomly allocated clusters are as shown in Figure 1. The study has been carefully designed to minimize the coefficient of variation among the clusters.

### Map of the study area

#### Study population

In this study, the study population will comprise high-CVD-risk patients stratified by the Framingham general CVD risk scoring system [18,19]. Only those patients who have a CVD risk score higher than 15% will be included.

### Eligibility criteria for individual patients to enroll in the study

Selection of participants will be according to the following inclusion and exclusion criteria.

#### Inclusion criteria

1. Diabetic patients with high CVD risk according to the Framingham general CVD risk score (>15%)

2. Hypertensive patients with high CVD risk according to the Framingham general CVD risk score (>15%)

3. Patients who are willing to participate in the study

#### Exclusion criteria

1. Any female patients who are pregnant or trying to become pregnant

2. Patients aged younger than 35 years

3. Patients with documented type I diabetes

4. Those on long-term steroid therapy (more than 2 weeks)

5. Those on long-term non-steroidal anti-inflammatory drugs (NSAIDs) (i.e., every day for at least 1 year)

6. Patients with cancer

7. Patients with known secondary hypertension such as primary aldosteronism, Cushing’s syndrome or pheochromocytoma

8. Patients with severe chronic pulmonary diseases using home oxygen therapy

9. Patients with chronic renal disease (creatinine ≧ 2.0 mg/dl)

10. Patients with congestive heart failure

11. Those with known previous diagnosis of CVD

### Ethical approval

The study protocol was approved by the Juntendo University Ethics Committee, Japan (no. 2011036) on 1 July 2011 and Chiang Rai Regional Hospital Ethics Committee, Thailand (no. CR0027.102/research/207) on 17 November 2011. The study has been registered as a current controlled trial internationally, and International Standard Randomized Controlled Trial Number Register ISRCTN39416277 was assigned on 3 January 2012 [20].

Participants will be well informed about the study process in their respective group of the study and will be free to decide to participate in the trial (Autonomy) [21]. There are no racial or ethnic criteria for inclusion in the study (Equity). Participants in both the control and intervention groups will receive routine health services and also some trial-related screening service benefits such as salt intake estimation, lipid profile, hemoglobin A1c and body composition measurements (Table 1). There will be no introduction of risk to the participants (Beneficence).

**Table 1:** Overview of data collection and measurements in both CRT arms

	**Follow-up**												
Visit number	1	2	3	4	5	6	7	8	9	10	11	12	13
Month	0	1	2	3	4	5	6	7	8	9	10	11	12
Inclusion and exclusion criteria	x												
Framingham score	x			x									x
Estimated 24-hour salt intake, overnight urine	x			x									x
Blood pressure	x	x	x	x	x	x	x	x	x	x	x	x	x
Body composition	x			x									x
Blood glucose	x												
HbA1c	x												
Lipid profile	x												
BUN, creatinine	x												
Sodium, potassium	x												
Uric acid	x												
CVD event	x			x									x
CVD death	x			x									x
Retinal caliber	x												
Demographic characteristics	x												
Questionnaires assessing behavioral change	x			x									x
Questionnaires assessing health costs	x			x									x

### Enrollment

**Figure 2:**
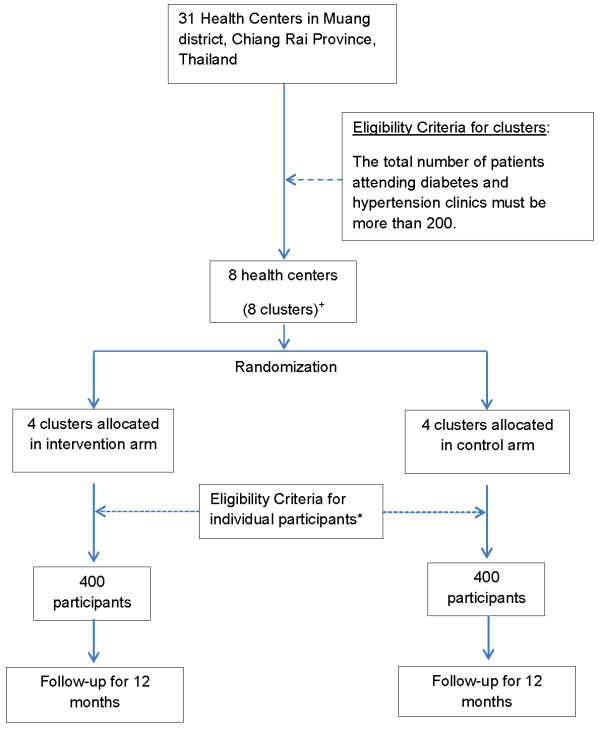
Flow chart for enrollment and follow-up plan for CRT.^*^The eligibility criteria refer to the inclusion and exclusion criteria under the heading of study population. ^+^Clusters are shown in Figure 1.

There are 31 health centers in Muang District. We will apply the criterion that there must be a minimum of 200 patients attending the diabetes and hypertension clinics in the health centers. (Figure 2)This selection criterion is set in order to obtain a sufficient number of high-CVD-risk patients with a high enough Framingham general CVD risk score (>15%) within the study site clusters. Using the existing provincial databases for screening, eight health centers meet this criterion.

#### Randomization and application of eligibility criteria

A statistician, blinded to the recoded number of health centers, will generate the random allocation sequence using Stata software, version 11 (College Station, TX, USA). Simple randomization will be used to allocate the eight clusters into intervention and control groups. (Figure 2)

#### Concealment of allocation

The study objectives will be clearly explained to the directors of the health centers, the cluster representatives. Informed consent and agreement to enroll in the study will be requested before random allocation into either arm of the study. Up until the point of enrollment, the researcher and cluster representative cannot know the allocation of the clusters. Only after the agreement to enroll, the enrollment and the random allocation will the procedures, applicable to their particular study group, be explained to the cluster representatives, and this will be done on different days.

At the allocated health center, participants will be recruited by fulfilling the eligibility criteria for individual enrollment. Written informed consent for trial participation will be requested of eligible participants. Likewise, the study participants will not know into which arm of the study they will be allocated until they have enrolled in the study. The participants will receive a thorough explanation of the procedures of the arm to which their attending health center has been randomly allocated. They will not be informed or will be unaware of the fact that there is another group in the study, nor will they be informed of the procedures of the other arm.

Each health center will enroll 100 participants. Chronology of the recruitment is as follows: When the patients come to the health center for their routine check-up, they will be invited to participate in the trial. On the next visit, the eligibility criteria will be checked, and informed consent will be obtained from those who are eligible. Then, the participants will receive a detailed explanation of the procedures of their respective study arm.

#### Blinding

The cluster representatives will know their allocated arm after random allocation, and the study procedure of only their allocated arm will be explained to them. Individual participants will be given information pertaining to the study group in their allocated cluster only after enrollment, and thus blinded. The nature of the study does not allow double blinding. However, the cluster randomized trial design will prevent contamination between the two arms of the study.

### Intervention

The two major components of the education program are the visualization tools and the special health education classes organized by the dietician. We hypothesized that after seeing both the amount of their daily dietary salt intake and the salt content in their daily food, people might be inspired to reduce dietary salt intake. Therefore, we will use visualization tools to inform the patients of their estimated 24-hour salt intake (Table 2).

**Table 2:** Visualization tools used in the intervention arm of the study

**Visible salt level measurements that participants can see**	**Equipment**	**Frequency of application**	
1. Salt intake in the last 24 hours	Measurement of sodium in overnight collected urine by KME-03	Every monthly visit to the health center	
2. Dietary salt content in daily food/ soup	Pocket Salt-meter PAL-ES2 Atago, Tokyo	On the 1- and 6-month visit	

### Visualizing salt content of food by digital hand-held “pocket” salt meter

**Figure 3:**
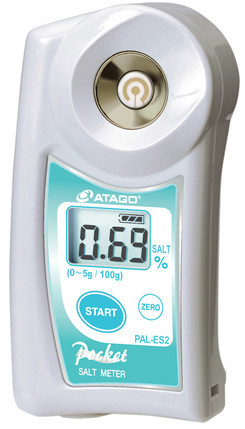
** Digital hand-held salt meter for measuring food salt content**[22].

Participants will be requested to bring their usual food (e.g., curries, soup and fried foods) to the health center. We will use the digital hand-held pocket PAL-ES2 salt meter (Atago Co., Ltd, Tokyo, Japan) to measure the salt concentrations in the participants’ food (Figure 3). The salt content will be shown in displayed units of g/100 ml [22]. This intervention informs the patients about the amount of salt in their food.

#### Revealing estimated daily salt intake

A KME-03 salinity checker (KOUNO ME Institute, Kanagawaken, Japan) will be used to determine the estimated daily salt intake [23]. During each monthly health center visit, participants in the intervention group will be informed of their 24-hour estimated salt intake tested from overnight collected urine. The monthly serial measurement of individual participants will be reported using an in-line graph documenting whether the salt intake is above or below 6 g /day.

#### Health education class

The small group education class will meet two times: the first, 1 month after enrollment and the second, 3 months after enrollment. The participants will be requested to bring their daily food to the health centers. The salt content in the home-made food such as soups, curries and sauces will be analyzed by a PAL-ES2 digital hand-held salt meter. The salt content in the processed food such as canned food, ready-to-eat food and instant noodles will be explained referring to the nutritional facts labels. The dangers related to a high-salt diet such as potential cardiovascular death, will be explained. There will be a taste test for participants to determine the saltiness of the sample food so that they can be made aware that individual tastes are subjectively different. After that, the dietician will explain how to reduce the daily dietary salt content based on the recommendations published by the Thailand Ministry of Public Health. These recommendations aim to reduce daily salt intake to less than 2,400 mg of sodium/day [24].

The group education by a dietician will take 2 hours. The education will focus on continuously motivating the participants by serial assessment results and providing assistance to reduce daily dietary salt intake. The dietician will identify the traditional high-salt foods such as fish sauce, processed pork, and other products, giving advice for alternative ways of cooking palatable food without high salt levels. Dietary sodium fact sheets will be provided for the patients to keep at home.

### Control group

Participants in the control arm will receive both routine health care services and health education programs. The measurement and assessment tests will also be provided to the participants in the control arm (Table 1). Participants in the control arm will receive routine health education, which is a brief individual education session not particularly focused on salt reduction. The differences between the two arms are (1) hypothesis-driven health educational interventions and (2) revealing of the results of the serial assessment tests, such as KME −03, to the control arm participants only at the end of the trial (Table 2).

### Definition

1 Salt Reduction

Salt reduction is defined as decreasing individual salt intake to less than 6 g/day, according to the Thailand Ministry of Public Health.

2 Diabetes

Diabetes mellitus is defined as either one of the following criteria: HbA1c concentration of more than 6.5% or fasting blood glucose of more than 126 mg/dl at least twice or an on the spot blood glucose level of more than 200 mg/dl at least once, simultaneously with 2-h post-prandial blood glucose of more than 200 mg/dl or history of taking oral hypoglycemic drugs [25,26].

3 Hypertension

Hypertension is defined as a systolic blood pressure (SBP) of ≥ 140 mmHg or a diastolic blood pressure (DBP) of ≥ 90 mmHg and higher in subjects who are not taking antihypertensive medication or subjects who are taking the medication for hypertension [27].

4 Cardiovascular diseases (CVD) events

Myocardial infarction, coronary insufficiency, unstable angina, ischemic stroke, hemorrhagic stroke, transient ischemic attack (TIA), peripheral artery disease or heart failure will be counted as CVD events in this study. Patients with a known diagnosis of CVD will be screened and excluded.

5 CVD mortality

This is defined as any documented death caused by CVD during the study period.

### Study outcome and outcome measurements

#### Primary outcome

Blood pressure measurements will be used as the primary outcome of the study. Blood pressure will be measured serially at baseline and on the 3-, 6- and 12-month follow-up visits of the trial (Table 3). It will assess the impact of salt reduction on cardiovascular health. The HEM-907 IntelliSense professional digital blood pressure monitor (Omron Healthcare Inc., Lake Forest, IL, USA) will be used to measure blood pressure (Table 3).

**Table 3:** Study outcome, outcome measurement and assessment methods

**Outcome**	**Assessment**	**Outcome measure**
Primary	Blood pressure	Omron HEM-907 IntelliSense professional digital blood pressure monitor
Secondary	Individual salt intake estimate/24-hour	Overnight urine collection and measurement of urine sodium using the KME-03 (average of 3 successive days’ measurements)
Clinical	CVD risk	General Framingham score 10-year risk
	CVD incidence	Diagnosis of each CVD event and CVD death
	Surrogate outcome	Retinal vessel caliber

### Secondary outcome

Individual salt intake will be monitored to assess the behavioral changes related to salt intake. Overnight collected urine will estimate the salt intake by a KME-03 intake salinity checker [28-30]. The mean value of 3 successive days’ measurements will be recorded and assessed as outcome measures at baseline and on the 3- and 12-month visits. The CVD risk assessment, CVD events and CVD-related deaths will be followed up (Table 3). The Framingham general CVD risk score will be measured at study entry and assessed again at the end of the study period to assess whether salt reduction decreases the CVD risk. CVD event diagnosis will be based on the physician’s decision by clinical and confirmatory tests for respective CVD events at regional- or district-level hospitals. CVD events will be counted whenever they are newly diagnosed among study participants. Deaths caused by CVD and confirmed by the hospital records will be counted as CVD deaths. A masked committee consisting of two physicians who do not know the participant’s study arm will confirm the CVD events. Also of note, the retinal vessels caliber measurement will be used as a surrogate outcome for a CVD risk assessment.

### Sample size

For a conventional randomized control trial, a total of 240 participants, randomizing 120 into each arm, will have enough power (90%) to detect the minimal difference of systolic blood pressure 130 (±20) and 120 (±20) mmHg, diastolic pressure 90 (±20) and 80 (±20) mmHg, difference in 24-hour estimated salt intake 10 (±5) and 8 (±4) g/day and CVD event rate of 0.15% and 0.08% between the control and intervention group with a 95% confidence interval [6,31]. The cluster randomized trial design used in RESIP-CVD might result in loss of power and reduced efficiency. In this cluster randomized trial, the number of participants in each cluster will be the same, and other variable factors such as geography, food tradition, race and dietary habits are assumed not to be different for delineating the intracluster coefficient. To compensate the design effect, the inflation factor has been calculated by the formula (*Deff* = 1 + (*m–1*)*ρ*) where *Deff* is inflation factor for design effect, *m* is the size of each cluster, which is 30 and *ρ* is the intracluster coefficient which is assumed to be 0.07. The sample required for a cluster randomized trial was inflated to 720. With a loss to follow-up of approximately 10%, we expect that the sample size of 800 patients will yield sufficient power to detect the desired minimal difference in primary and secondary outcomes between the intervention and control group. All eight clusters of the RESIP-CVD study will enroll 100 eligible participants.

We identified the confounders and planned to collect the information for multivariate analysis adjustment. (Table 4)

**Table 4:** Confounder measurements

**Confounder**	**Variables**	**How to measure**
Demographic characteristics	Age, sex, social status, family size, educational attainment, occupational history, income	Questionnaire
Lifestyle	Smoking, alcohol	Questionnaire
Genetic factor	Family history of diabetes/hypertension	Questionnaire
Drugs	Different medications for diabetes and hypertension	Clinical record

### Data collection and follow-up

Before starting data collection, we completed feasibility surveys at the study site clusters. A pilot intervention was tested at a non-eligible health center. The timeline of the data collection and assessment will be formatted as seen in Table 1.

### Baseline survey

In this CRT, we wanted to test the health intervention in high-CVD-risk patients. A low incidence of CVD events in low-CVD-risk patients may warrant a larger sample size than that we calculated and a longer follow-up than we can perform. Therefore, we will recruit only the high-CVD-risk patients stratified by using the Framingham general CVD risk scoring system (10-year risk). Calculation of the Framingham score will require information such as age, sex, smoking status, systolic blood pressure, history of taking antihypertensive drugs, history of diabetes and body mass index (BMI).

After recruitment, the Framingham score of the eligible patients will be assessed in a baseline survey [19,20]. The risk score calculator in formula excel spread sheet published by the Framingham Heart Study will be distributed to the study site HCs [32]. HC staffs will be trained to apply it for the CVD risk stratification.

### Follow-up plan

The staffs of the health centers will be reinforced with additional staff from the research project. The research team will call patients who failed to visit the health center for the assessment. The research nurse in charge will visit the patient’s house and collect the urine sample if the participants are unable to come to the health center on the appointed date for assessment. The control arm participants will not have measurements as frequently as the intervention group participants. At the health center level, nurses are the main persons to follow up the patients and deliver the drugs. Patients are usually referred to the district hospital or regional hospital whenever there is poor control of their disease or they have problems such as a poor control of BP or blood glucose level or any symptoms suggestive of CVD. We will use this routine health system process to strengthen the follow-up.

### Analysis plan

**Figure 4:**
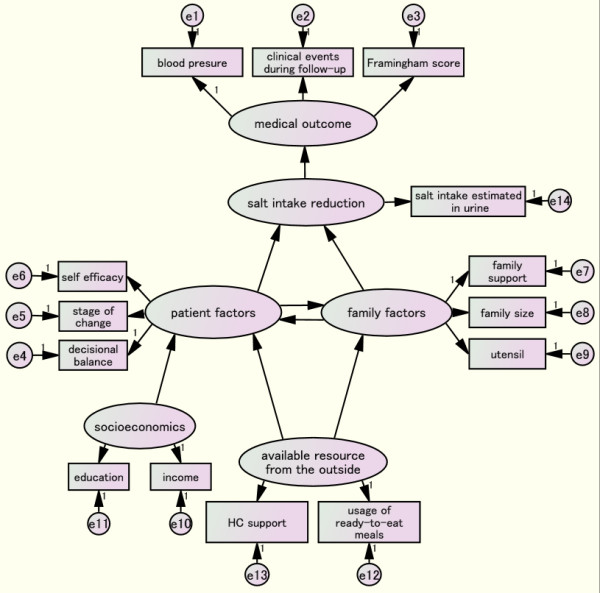
Path model to analyze predictors of behavioral change by structural equation modeling (SEM).

The intention-to-treat analysis will be followed. The mean blood pressure and mean daily salt intake of both the intervention and control arms will be compared at the individual level at the 1-year follow-up using Student’s *t* test or the non-parametric test based on distribution. The analysis will be carried out at both the cluster and individual levels. The CVD incidence rate will be compared by time-to-event analysis. A structural equation modeling analysis will be used to determine the behavioral change predictors. Information such as the self-efficacy, stage of attitudinal change, decisional balance, family support, health center support, ready-made meals usage and cooking utensils will be collected by questionnaires and analyzed by the SEM (Figure 4).

Differences in CVD events and deaths and the associated costs between intervention and control groups will be estimated for up to 10 years after the intervention. Lifetime costs and health outcomes for our study population (aged 35 years and above) will be estimated using data both in and outside of Thailand. Outcomes of the analysis will be: CVD events and deaths averted and the cost over 10 years, incremental cost-effectiveness ratio (ICER) per CVD death averted, lifetime costs and quality-adjusted life years (QALYs) gained and ICER per QALYs gained. The analysis will be performed using a societal perspective. All the future costs and outcomes will be discounted at a rate of 3% per annum. Sensitivity analysis will be employed to check the robustness of the results. Budget impact analysis will be applied. From a government perspective, the direct medical cost of CVDs will be estimated both with and without special health education in Chiang Rai Province in the course of the next 10 years in order to assess its impact on the Universal Health Coverage Scheme budget.

## Discussion

There have been many previous studies related to salt reduction interventions [33]. This current study is unique as the study population is exclusively composed of high-CVD-risk patients stratified by applying the universal Framingham general CVD risk scoring system. Therefore, the intervention can be prioritized, focusing on the high-risk individuals most vulnerable to CVD morbidity and mortality at the community level within a developing setting. The intervention will not merely be a case of counseling and providing information, however. The intervention group will be able to clearly see and understand the salt level content in their daily food and their estimated daily salt intake following urinary salt measurement. Furthermore, we will use equipment which is affordable within developing settings to visualize the salt content in food and the patient’s urine. The effectiveness of the salt reduction intervention will be assessed by multiple outcomes (Table 3).

In many previous clinical studies, reduced salt intake has been shown by means of urinary sodium measurement in urine collected over a period of 24-hours [8,34]. In the current study, overnight urine will be collected and assessed for urinary sodium [23,35]. This is a more feasible and practicable outcome measurement that overcomes the possible bias caused by under-collection of urine. In addition, we will assess the blood pressure changes comparatively between participants in the intervention and control groups. The reported incidence of CVD events and deaths will be compared to determine the clinical effectiveness of the health promotion intervention.

Health system costs and reimbursement systems differ between developing and developed countries. Thailand is a middle-income country in Southeast Asia, with 100% insurance coverage for non-communicable disease-related services. It is estimated that by allocating just US$ 0.06 per person for voluntary salt reduction, 310,000 deaths can be prevented over a 10-year period [36,37]. WHO has recommended salt reduction in addition to risk modification by advocating multiple-drug treatments using, for example, anti-hypertensive, oral hypoglycemic agents and lipid-lowering medications. It is necessary to determine whether the prevention of CVD incidence by a salt reduction strategy is cost-effective in terms of treatment and health service costs compared to traditional drug treatment that has little concern for lifestyle modification [38]. The cost-effectiveness analysis of our study is expected to answer these questions for developing countries.

We have designed the study to have minimal residual confounding factors and selected practical and contemporary approaches that overcome hard-to-achieve lifestyle modifications. Thus, we expect that implementing a health promotion intervention trial in a practical setting will yield comprehensive and applicable evidence for global reduction of CVD deaths and prevention of CVD events.

### Trial status

Recruiting participants.

## Abbreviations

CRT: Cluster randomized trial; HC: Health center; FMH scoring: Framingham general CVD risk scoring; CVD: Cardiovascular disease; NSAID: Nonsteroidal antiinflammatory drug; SEM: Structural equation modeling; ICER: Incremental cost-effectiveness ratio; QALYs: Quality-adjusted life years.

## Competing interests

The authors declare that they have no competing interests.

## Authors’ contributions

MNA and MY were the lead authors in charge of study design. SM, HY, TK, KM, ST and HF provided specific advice and relevant information to design the study protocol. YH and KO were in charge of retinal arterial caliber measurement. SN, SK and EM also provided advice and comments for this study. MNA wrote the manuscript and finalized the article. All authors read and approved the final manuscript.
